# QuEChERS-同位素内标-高效液相色谱-串联质谱法测定动物源性食品中植物生长调节剂类农药残留

**DOI:** 10.3724/SP.J.1123.2021.01029

**Published:** 2021-11-08

**Authors:** Wei DAI, Qiao LI, Ming ZHU, Yixin LIANG, Qiu CAI, Mian WANG, Jie LI, Kangshu LIU, Xingning WANG

**Affiliations:** 贵阳海关综合技术中心, 贵州 贵阳 550081; Comprehensive Technology Centre of Guiyang Customs, Guiyang 550081, China; 贵阳海关综合技术中心, 贵州 贵阳 550081; Comprehensive Technology Centre of Guiyang Customs, Guiyang 550081, China; 贵阳海关综合技术中心, 贵州 贵阳 550081; Comprehensive Technology Centre of Guiyang Customs, Guiyang 550081, China; 贵阳海关综合技术中心, 贵州 贵阳 550081; Comprehensive Technology Centre of Guiyang Customs, Guiyang 550081, China; 贵阳海关综合技术中心, 贵州 贵阳 550081; Comprehensive Technology Centre of Guiyang Customs, Guiyang 550081, China; 贵阳海关综合技术中心, 贵州 贵阳 550081; Comprehensive Technology Centre of Guiyang Customs, Guiyang 550081, China; 贵阳海关综合技术中心, 贵州 贵阳 550081; Comprehensive Technology Centre of Guiyang Customs, Guiyang 550081, China; 贵阳海关综合技术中心, 贵州 贵阳 550081; Comprehensive Technology Centre of Guiyang Customs, Guiyang 550081, China; 贵阳海关综合技术中心, 贵州 贵阳 550081; Comprehensive Technology Centre of Guiyang Customs, Guiyang 550081, China

**Keywords:** 高效液相色谱-串联质谱, QuEChERS, 植物生长调节剂, 矮壮素, 噻苯隆, 多效唑, 动物源性食品, high performance liquid chromatography-tandem mass spectrometry (HPLC-MS/MS), QuEChERS, plant growth regulators, chlormequat chloride, thidiazuron, paclobutrazol, animal derived foods

## Abstract

建立了高效液相色谱-串联质谱法测定动物源性食品中植物生长调节剂类农药残留量的方法。选取猪肉、牛肉、鸡肉、猪肝、鸡蛋和牛奶作为样品,样品经乙腈提取,4 g无水硫酸镁(MgSO_4_)和1 g氯化钠(NaCl)盐析脱水后,取上清液经50 mg *N*-丙基乙二胺(PSA)+50 mg十八烷基硅烷(C18)粉末净化(含150 mg MgSO_4_)。采用Agilent ZORBAX Eclipse Plus C18柱分离待测物,电喷雾电离,正负离子切换多反应检测模式检测,以乙腈和5 mmol/L乙酸铵水溶液作为流动相进行梯度洗脱,基质匹配内标法定量。在猪肝、鸡蛋基质中,矮壮素、噻苯隆和多效唑在0.1~100 μg/L范围内线性关系良好;在猪肉、牛肉和鸡肉中3种植物生长调节剂在0.1~50 μg/L范围内线性关系良好;在牛奶基质中,噻苯隆和多效唑的线性范围为0.05~10 μg/L,矮壮素的线性范围为0.05~5 μg/L,相关系数(*r*^2^)均大于0.990。以信噪比(*S/N*)≥3对应的添加水平作为检出限(LOD), *S/N*≥10对应的添加水平作为定量限(LOQ),矮壮素、噻苯隆和多效唑在不同基质下的LOD为0.01~0.1 μg/kg, LOQ为0.5~5 μg/kg。分别添加LOQ、2倍LOQ和10倍LOQ 3个水平的目标化合物,平均回收率为70.0%~117.4%, RSD为0.8%~16.1%。该方法操作简单、灵敏度高,采用基质匹配内标法定量,能最大限度地消除基质干扰,使检测结果更加精确,可满足动物源性食品中矮壮素、噻苯隆和多效唑残留的定量检测工作。

## Abstract (en)

As among the most widely used pesticides in agriculture, plant growth regulators (PGRs) have a positive influence on plants. However, the overuse of PGRs may induce toxicity in food and even be hazardous to human health. Numerous studies have investigated the presence and residues of PGRs on vegetables and fruits. Animal-derived foods are one of the most dominant food sources providing nutrients to fulfil the daily dietary intake, and could also be potentially contaminated by PGRs. However, there is little information on PGR residues in animal-derived foods. Standardization also lacks among the techniques for PGR determination in animal-derived foods, thereby restricting the further establishment of pesticide usage and food safety regulations. Therefore, in this study, a rapid and effective method for analyzing chlormequat chloride, thidiazuron, and paclobutrazol in animal-derived food samples was established. The method primarily involves high performance liquid chromatography-tandem mass spectrometry combined with the use of isotope-labeled internal standards. The extraction and clean-up procedures were based on the QuEChERS method. The analytes were extracted from pork, beef, chicken, pork liver, egg, and milk samples using acetonitrile, followed by 4 g anhydrous magnesium sulfate (MgSO_4_), and 1 g sodium chloride (NaCl). The supernatant was removed using a mixture of 50 mg *N*-propyl ethylenediamine (PSA), 50 mg octadecyl silane (C18), and 150 mg MgSO_4_, and then passed through a 0.22 μm membrane filter before determination. The Agilent ZORBAX Eclipse Plus C18 column (150 mm×3.0 mm, 1.8 μm) was used to separate the analytes under a gradient elution program, with acetonitrile and 5 mmol/L ammonium acetate solution as mobile phases. The analytes were detected by mass spectrometry using the positive and negative electrospray ionization modes under the multiple reaction monitoring mode. Matrix-matched calibration combined with internal standards was used to quantify the PGRs. The linear regression correlation coefficients (*r*^2^) for the PGRs were all greater than 0.990 in the corresponding linear concentration ranges. Chlormequat chloride, thidiazuron, and paclobutrazol showed good linearities in the range of 0.1-100 μg/L for the egg and pork liver samples and 0.1-50 μg/L for the pork, beef, and chicken samples. For the milk samples, thidiazuron and paclobutrazol showed good linearities in the range of 0.05-10 μg/L, while chlormequat chloride showed linearity in the range of 0.05-5 μg/L. The limit of detection (LOD) and limit of quantification (LOQ) for each PGR were based on the signal-to-noise (*S/N*) ratios. Under optimal conditions, the LODs ranged from 0.01 μg/kg to 0.1 μg/kg, where the LOD was defined as the amount of the tested compound that generated an *S/N* ratio higher than 3. In addition, the LOQs were in the range of 0.5-5 μg/kg, with an *S/N* ratio higher than 10. The precision and accuracy were evaluated by recovery experiments. At the LOQ, twice the LOQ, and 10 times the LOQ, the mean recoveries were in the range of 70.0%-117.4%, and the relative standard deviations (RSDs) ranged from 0.8% to 16.1%. The results indicated that the proposed method is accurate and reliable. This method is a modification of the QuEChERS method, and is advantageous owing to its simplicity and high sensitivity. The use of matrix-matching calibration curves and internal standards can eliminate matrix interference, thereby increasing the accuracy of the method. This method satisfies the testing requirements for chlormequat chloride, thidiazuron, and paclobutrazol residues in animal-derived foods, and is promising for the determination of other PGRs or other types of pesticides in animal-derived foods.

植物生长调节剂(plant growth regulators, PGRs)是一类广泛应用于现代农业的植物激素化合物,分为天然植物激素和人工合成两类,可以促进、抑制或延缓植物的生长,对植物的生长具有调节作用^[1,2]^。在农业科技高速发展的今天,植物生长调节剂在提高农作物产量、改善农作物品质方面发挥着重要作用。农作物作为畜禽动物的主要饲料,过多或不当的使用植物生长调节剂,会使残留的农药聚集在动物体内,不易排出,最后通过食物链进入人体,长期食用会对人体健康造成潜在危害^[3,4,5,6]^。矮壮素、噻苯隆和多效唑是目前我国广泛使用的植物生长调节剂,可作用于蔬菜、果树、水稻、小麦、烟草、棉花等作物^[7,8]^,它们在帮助提高农作物产量的同时也存在着一定的潜在风险。肖泳等^[9]^在对国内市售的动物源性食品的检测中发现,鸡蛋和牛奶中均有矮壮素残留且检出率占比较高。对外出口方面,自2003年以来,我国出口的食品中矮壮素和多效唑多次因残留问题被欧盟、日本、澳大利亚等国家发布预警通告,对我国食品的出口造成了一定影响。目前国内外对矮壮素、噻苯隆和多效唑农药残留的研究主要针对蔬菜、水果、粮油和药材,对动物源性食品中的研究较少。因此本工作选择矮壮素、噻苯隆和多效唑作为研究对象,建立动物源食品中植物生长调节剂的检测方法,对今后动物源性食品监管以及进出口风险监控具有一定的意义。

目前欧盟、日本及澳大利亚对于矮壮素、噻苯隆和多效唑的使用都制定了相应的最大残留限量(MRL),我国最新版GB 2763-2019《食品安全国家标准食品中农药最大残留限量》^[10]^涵盖了19种植物生长调节剂,其中对这3种PGRs也给出了相应的限量规定,详见表1。欧盟法规(EC) NO 396/2005中规定,在动植物源食品中若未规定限量的农药,则按0.01 mg/kg来执行^[11]^。同样地,日本2006年实施的“肯定列表制度”中规定,对于未制定MRL或不是豁免物质的农业化学品,在食品中需遵从“一律标准”规定,即不得超过0.01 mg/kg^[14]^。

**表1:** 不同国家对3种植物生长调节剂的MRL规定

Analyte	Products	China^[10]^/(mg/kg)	EU^[11]^/(mg/kg)	Japan^[12]^/(mg/kg)	Australia^[13]^/(mg/kg)
Chlormequat	mammals (muscle)	0.2^*^	0.3	0.2	0.2
chloride	mammals (liver)	0.1^*^	1.5	0.1	0.5
	poultry (muscle)	0.04^*^	0.05	0.04	0.05
	eggs	0.1^*^	0.15	0.1	0.1
	milk	0.5^*^	0.5	0.5	0.5
Thidiazuron	mammals (muscle)	-	0.01	0.1	0.05
	mammals (liver)	-	0.01	0.1	0.05
	poultry (muscle)	-	0.01	0.2	-
	eggs	-	0.01	0.1	-
	milk	-	0.01	0.03	0.01
Paclobutrazol	mammals (muscle)	-	0.01	0.01	-
	mammals (liver)	-	0.01	0.01	-
	Poultry (muscle)	-	0.01	0.01	-
	eggs	-	0.01	0.01	-
	milk	-	0.01	0.01	-

* Temporary MRL; -: unspecified.

我国2009年实施的国家标准GB/T 20772-2008^[15]^规定了动物肌肉中461种农药及相关化学品残留量的液相色谱-串联质谱检测方法,该标准对动物肌肉中农药残留的研究有着重要的指导作用。GB/T 20772-2008采用凝胶渗透色谱法(GPC)作为前处理方法对脂肪含量较高的动物肌肉进行净化分离,该方法作为动物源性食品的主要净化手段,具有很好的净化效果,但GPC方法操作相对复杂,不适用于大批量样品的检测工作。目前国内外对于动物源食品中农药残留的前处理方法除了GPC法外,还有固相萃取法(SPE)、分散固相萃取(d-SPE)和QuEChERS法等。SPE法净化效果好且操作简单,但由于净化方法单一,难以满足同时净化理化性质差异较大的化合物^[16]^。QuEChERS法是由Anastassiades等^[17]^于2003年研发的一个新型分散固相萃取技术,通过盐析分层后,利用基质分散萃取原理,加入吸附剂填料以去除大部分的基质干扰物(如色素、糖、脂肪、有机酸等),具有快速、简单、经济、高效、可靠和安全等优点^[18,19]^,如今已经广泛应用于食品的检测工作中。在近些年的发展过程中,QuEChERS法应用已经从蔬菜水果这类相对简单的基质扩展到其他更为复杂的基质上,如:动物源性食品、乳制品、茶叶等。Oliveria等^[20]^、Kao等^[21]^建立了QuEChERS法检测畜禽肉中的农药残留,但由于部分脂类物质与乙腈是共萃物导致QuEChERS法提取效率不高,因此使用内标十分必要^[18,22]^。内标法可以在很大程度上消除提取和净化带来的损失以及基质效应的干扰,使得定量结果更加准确。

高效液相色谱-串联质谱(HPLC-MS/MS)是如今最常用的多农残检测方法之一,相较于其他检测方法,如气相色谱法(GC)、气相色谱-串联质谱法(GC-MS/MS)、高效液相色谱法(HPLC), HPLC-MS/MS具有灵敏度高、特异性强、选择性好等特点且前处理无需衍生^[1,8,23]^。本工作通过优化QuEChERS的前处理方法,采用HPLC-MS/MS的检测手段,结合同位素内标法定量,建立了一个快速检测动物源性食品中3种PGRs残留的分析方法,该方法操作简单、灵敏度高,能够精准定量,适用于大批量动物源性食品中PGRs残留的检测。

## 1 实验部分

### 1.1 仪器设备

Agilent 1290 InfinityⅡ-6470高效液相色谱-三重四极杆质谱联用仪,配Agilent Jet Stream电喷雾离子源(AJS ESI)(美国Agilent公司);离心机(美国Beckman Coulter公司);涡旋振荡器(德国Heidolph公司); Milli Q超纯水系统(美国Millipore公司)。

### 1.2 材料与试剂

矮壮素(100 mg/L, CAS: 999-81-5,溶剂为异丙醇,农业部环境保护科研检测所);多效唑(100 mg/L, CAS: 76738-62-8)和噻苯隆(100 mg/L, CAS: 51707-55-2)(溶剂为甲醇,北京坛墨质检科技有限公司);矮壮素-D_9_、噻苯隆-^13^C_6_(上海甄准生物科技有限公司);多效唑-^15^N_3_(上海安谱科技股份有限公司);乙腈、甲醇(色谱纯,德国Merck公司);氯化钠(山东西亚化学工业有限公司);无水硫酸镁(国药集团化学试剂有限公司);乙酸铵(色谱纯,天津市科密欧化学试剂有限公司); *N*-丙基乙二胺(PSA)(天津博纳艾杰尔科技有限公司);十八烷基硅烷(C18)(上海安谱科技股份有限公司)。

### 1.3 溶液的配制

内标储备液(100 mg/L):分别准确称取矮壮素-D_9_、噻苯隆-^13^C_6_、多效唑-^15^N_3_适量,用甲醇配制成质量浓度为100 mg/L的标准储备液,于-20 ℃储存备用,有效期6个月。

混合内标使用液(1 mg/L):分别准确吸取3种内标储备液100 μL于10 mL容量瓶中,用甲醇定容,得到质量浓度为1 mg/L的混合内标使用液,于0-5 ℃保存,有效期2周。

混合标准使用液(1 mg/L):分别准确吸取质量浓度为100 mg/L的矮壮素、噻苯隆和多效唑标准品100 μL,氮吹干后用甲醇定容至10 mL,得到质量浓度为1 mg/L的混合标准使用液,于0-5 ℃保存,有效期2周。

基质混合标准工作溶液的配制:分别准确吸取适量混合标准使用液和混合内标使用液,用空白样品基质(猪肉、牛肉、鸡肉、猪肝、鸡蛋和牛奶)溶液配制成不同浓度的基质混合标准工作溶液(含混合内标使用液10 μg/L)。

### 1.4 样品的前处理

本工作所用样品均为市售。

称取样品5.00 g(牛奶样品2.00 g)于50 mL离心管中,加入100 μL质量浓度为1 mg/L的混合内标使用液,再加入9.9 mL乙腈,放入均质子涡旋振荡5 min,加入4 g无水硫酸镁、1 g氯化钠振荡5 min, 10000 r/min离心5 min。取上清液至装有50 mg PSA、50 mg C18和150 mg MgSO_4_的净化管中,充分振荡混匀,10000 r/min离心5 min,取上清液过0.22 μm滤膜,上机测定。

### 1.5 仪器分析条件

1.5.1 色谱条件

色谱柱:Agilent ZORBAX Eclipse Plus C18(150 mm×3.0 mm, 1.8 μm);柱温:35 ℃;流动相:A相为5 mmol/L乙酸铵水溶液,B相为乙腈;梯度洗脱程序:0~0.5 min, 10%B; 0.5~4 min, 10%B~90%B; 4~7 min, 90%B; 7.1 min, 10%B; 7.1~9 min, 10%B。流速:0.3 mL/min;进样量:5 μL。

1.5.2 质谱条件

采用AJS电喷雾离子源,正负离子切换多反应检测模式(MRM),干燥气温度:300 ℃;干燥气流速:10 L/min;鞘气温度:350 ℃;鞘气流速:11 L/min;雾化气压力:0.3 MPa;毛细管电压:3500 V(正、负模式);喷嘴电压:500 V(正、负模式)。3种植物生长调节剂及其内标的母离子、定量离子、定性离子、碰撞能量等见表2。

**表2:** 3种植物生长调节剂及其内标的质谱参数

Analyte	ESI	Parent ion (m/z)	Product ion (m/z)	Collisiom energy/eV	Fragmentor/V
Chlormequat cation	+	122.1	58.1*	35	100
			63.0	35	
Thidiazuron	-	219	70.9	35	80
			100.0*	15	
Paclobutrazol	+	294.1	57.2	15	100
			70.0*	20	
			124.9	25	
Chlormequat-D_9_ cation	+	131	66.1*	35	100
			68.2	25	
Thidiazuron-^13^C_6_	-	225	71.1	35	100
			100.1*	15	
Paclobutrazol-^15^N_3_	+	297.0	73.1*	25	120
			125.0	25	

*Quantitative ion.

## 2 结果与讨论

### 2.1 质谱条件的优化

以单针进样的方式,对质量浓度为1 mg/L的目标化合物进行分析优化。在目标物进入一级质谱后(矮壮素及其同位素内标以阳离子形式存在),均可产生稳定的[M+H]^+^离子,其中噻苯隆和噻苯隆-^13^C_6_也可产生稳定的[M-H]^-^离子,在比较响应值后发现,负模式下噻苯隆的响应较好,综合考虑后决定噻苯隆及其内标噻苯隆-^13^C_6_采用负离子模式(ESI^-^)进行一级质谱扫描,其余目标化合物采用正离子模式(ESI^+^)进行一级质谱扫描。在确定好化合物的母离子后,采用SIM模式对碎裂电压(fragmentor)进行优化。之后对母离子进行二级质谱扫描,在Product Ion模式下通过不断改变碰撞能量,得到效果最优的离子对。3种目标化合物及其内标的二级质谱图见图1。

**图1:**
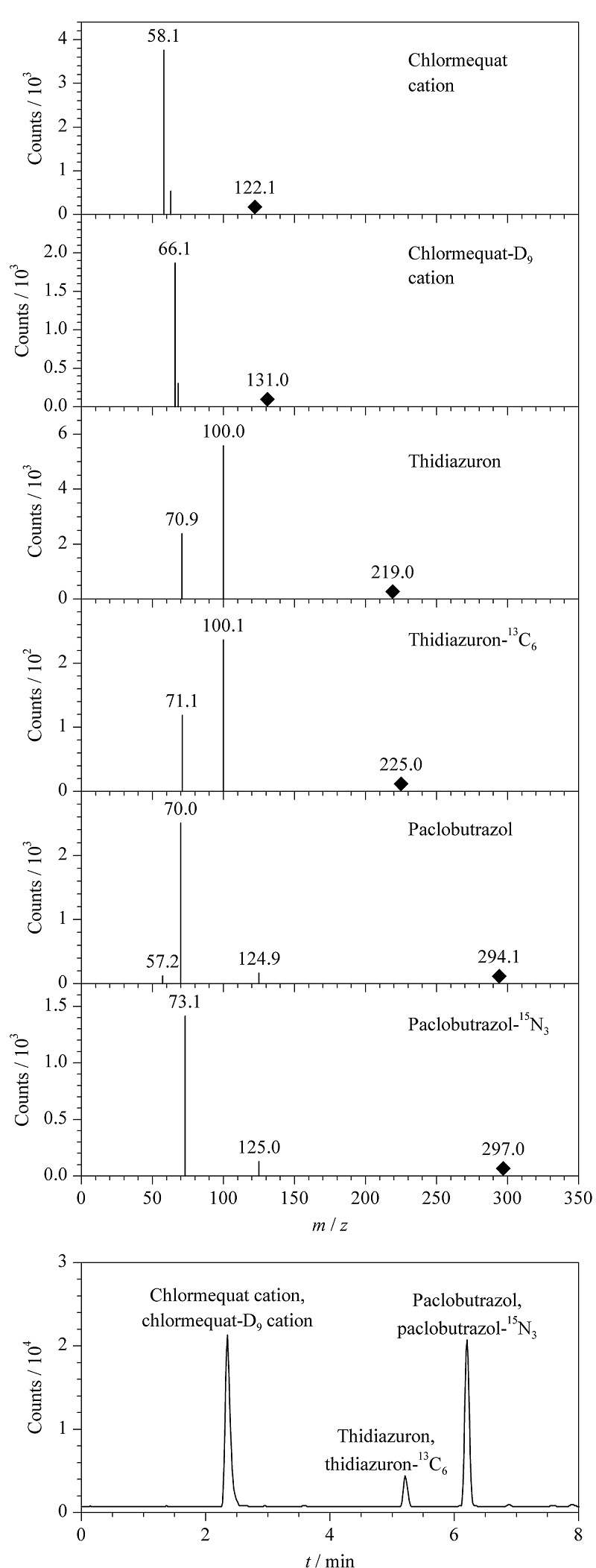
3种植物生长调节剂及其同位素内标的二级质谱图 及总离子流图

### 2.2 色谱条件的优化

2.2.1 色谱柱的选择

本实验对比了Hypersil GOLD C18(100 mm×2.1 mm, 1.9 μm)、Waters ACQUITY UPLC HSS T3(100 mm×2.1 mm, 1.8 μm)、Waters ACQUITY UPLC BEH C18(100 mm×2.1 mm, 1.8 μm)和Agilent ZORBAX Eclipse Plus C18(150 mm×3.0 mm, 1.8 μm)4种色谱柱对目标化合物的分离效果。几种化合物在这4种色谱柱上均能较好分离,但由于矮壮素极性较强,普通反相色谱柱对强极性化合物的保留比较弱^[1]^,在Hypersil GOLD C18、Waters HSS T3和Waters BEH C18上的保留时间均小于1 min,保留时间过短可能会受到基质峰的干扰,而在Agilent ZORBAX Eclipse Plus C18柱上,因为色谱柱较长,强极性化合物的保留时间大于2 min,各组分离子提取效果也比较理想,因此选择Agilent ZORBAX Eclipse Plus C18柱进行分析。

2.2.2 流动相的选择

在HPLC-MS/MS分析中,由于质谱采用正负切换MRM模式,流动相的改变对离子的电离情况、色谱峰峰形以及保留时间都有一定的影响^[24]^。通常选择甲醇或乙腈作为有机相,水相选择了纯水、0.1%(v/v)甲酸水溶液、5 mmol/L乙酸铵水溶液以及5 mmol/L乙酸铵+0.05%(v/v)甲酸水溶液进行比较。

实验表明,有机相中乙腈的分离效果优于甲醇,故选择乙腈作为有机相。水相中用水作为流动相,矮壮素的色谱峰出现拖尾现象,流动相中加适量缓冲盐或酸可以有效改善峰形^[25]^;当0.1%甲酸溶液和5 mmol/L乙酸铵+0.05%甲酸水溶液作为流动相时,所有待测组分峰均有较好的峰形,但响应值没有5 mmol/L乙酸铵水溶液作为流动相时高,可能是因为甲酸会使ESI^-^模式化合物电离受到抑制。因此选用5 mmol/L乙酸铵水溶液作为水相,所有待测组分的响应值均为最高且峰形较好。3种植物生长调节剂的总离子流图见图1。

### 2.3 提取溶剂的选择

植物生长调节剂类农药常用的提取溶剂是乙腈、甲醇和丙酮。根据样品性质,动物源食品的蛋白质和脂肪含量较高,与甲醇相比乙腈有更好的除蛋白的效果,且受pH影响较小^[26,27]^,因此本实验采用乙腈作为提取溶剂。以猪肉为基质样品,添加10 μg/kg的PGRs混合标准溶液,结合国内外现有的研究,选择乙腈、1%(v/v)甲酸乙腈、2%(v/v)甲酸乙腈、1%(v/v)乙酸乙腈和正己烷饱和的乙腈作为提取溶剂进行对比,如图2所示,当乙腈作为提取溶剂时,3种目标物的提取效率相对较好,回收率均大于60%,且乙腈提取时,多效唑的提取效率较高,因此选择乙腈作为提取试剂。

**图2:**
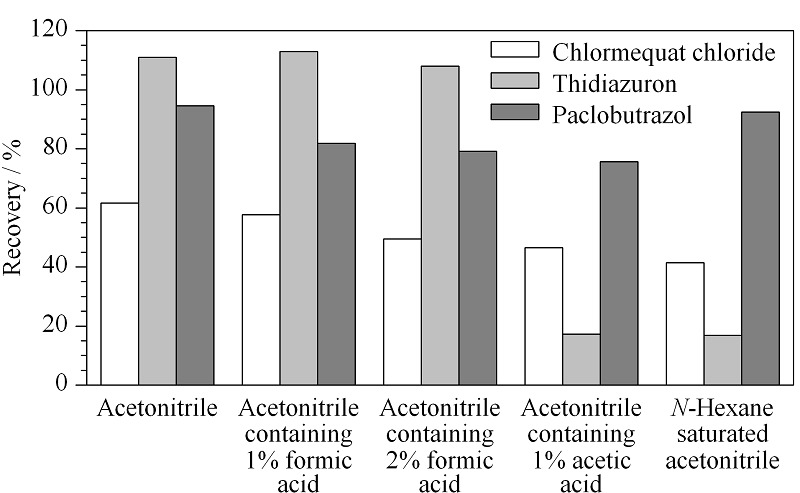
3种植物生长调节剂在不同提取溶剂时的提取效率

### 2.4 脱水剂和净化剂的选择

由于动物源性样品脂肪和蛋白质含量高,因此要对现有的QuEChERS方法进行优化,根据基质高脂肪、高蛋白的特点,对脱水剂和净化剂进行不同配比,从而达到一个最优的提取净化效果。

目前QuEChERS方法中常用的净化填料有PSA、C18和GCB^[24]^。PSA可以有效地去除糖、脂肪酸、有机酸、脂类和一些色素,C18能有效去除油脂等非极性杂质^[25]^, GCB去除叶绿素和色素效果明显^[1]^。此外还有许多新型填料被研发并运用于市场中,如Agilent公司的EMR-Liquid增强型基质去除吸附剂,对脂质吸附力强,有较好的去脂效果^[4]^。取1.5 mL质量浓度为10 μg/L的混合标准液,分别加入装有PSA、C18、PSA+C18和PSA+C18+GCB的净化管中,试验发现当含有GCB时,噻苯隆的回收率低,说明GCB对噻苯隆有吸附作用,影响实验的回收率。

因此,本工作以猪肉作为基质,添加10 μg/kg的PGRs标准溶液,按照1.4节的前处理步骤,结合国内外动物源性食品中常用的盐析脱水方法^[28,29,30]^,考察了不同脱水剂配比下目标化合物的提取效率(见图3)。对于3种PGRs来说,方法1(1 g NaCl+4 g MgSO_4_)即能到达较好的除水和盐析的作用,可以较好地将被分析物从水相分配到有机相;在选用方法1作为脱水剂后,再对100 mg PSA、100 mg C18、50 mg PSA+50 mg C18(均含150 mg MgSO_4_)以及EMR-Liquid的净化效果进行对比,结果见图3。结果显示,4种净化方法对噻苯隆和多效唑均有较好的提取效率;矮壮素的回收率相对较低,是因为矮壮素属于强极性化合物,强极性的化合物在萃取时,有机相的分配较差,导致回收率偏低^[18]^; EMR-Liquid作为常用的去脂净化剂,对多效唑和噻苯隆有着较好的提取效果,但对矮壮素的提取效果并不是很理想;噻苯隆在含有C18的净化条件时有较高回收率,回收率均大于90%,而PSA相较于C18有更好的去色素能力。综合考虑下本工作采用50 mg PSA+50 mg C18(含150 mg MgSO_4_)作为净化填料。

**图3:**
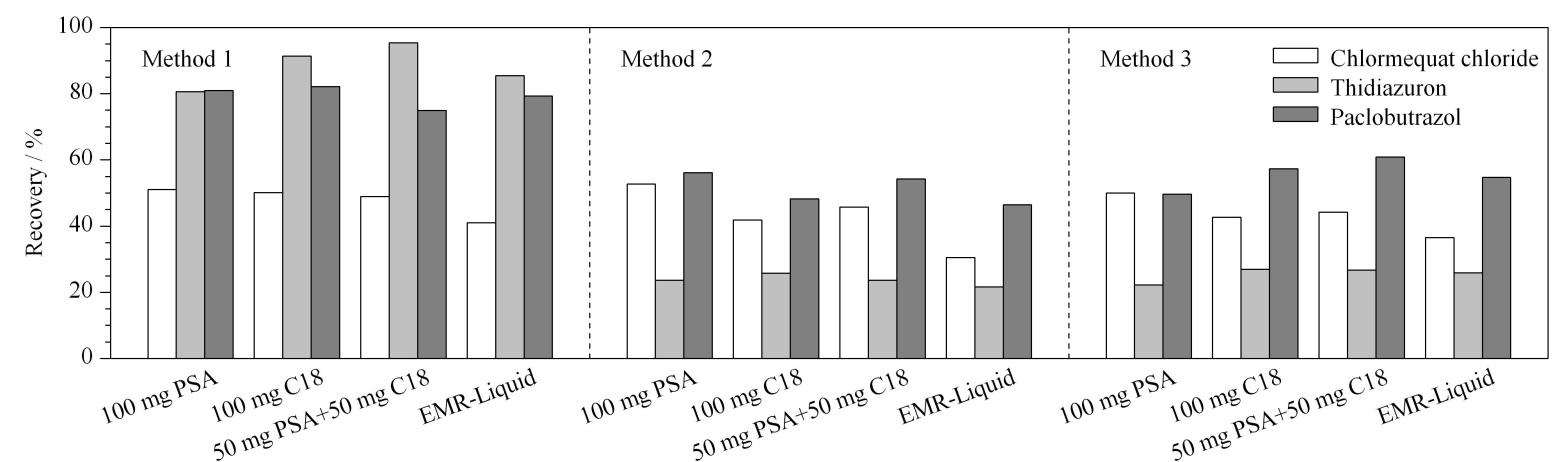
使用不同脱水剂及不同净化剂对3种植物生长调节剂回收率的影响(*n*=3)

### 2.5 基质效应

在HPLC-MS/MS检测复杂样品时,通常会受到基质的干扰,影响目标物的离子化,产生增强或抑制效应,这种现象称为基质效应(matrix effect, ME)^[1,22]^,干扰较大则会影响定量的准确性。因此,评价不同基质产生的基质效应十分必要。按照相对响应值法,用乙腈配制10 μg/L的混合标准溶液,测得峰面积为*A*,用猪肉、牛肉、鸡肉、猪肝、鸡蛋和牛奶作为基质按照上述1.4节的方法进行前处理,所得的空白基质分别用来配制10 μg/L的基质匹配混合标准溶液,测得平均峰面积为*B*,将所得峰面积代入以下计算公式:ME=*B/A*。若比值大于1为增强效应,比值小于1为抑制效应,当比值在(100±20)%(即0.8~1.2)时,通常认为基质效应弱^[18,31]^。表3结果表明,矮壮素和噻苯隆受到的基质干扰相对较小,但多效唑在不同的基质中受到的基质干扰均较强,除牛奶基质外,其余样品的基质效应均超过50%,尤其是在猪肝中,多效唑受到很强的抑制效应,这可能是因为肝脏基质比较复杂,新鲜肝脏中所含的色素、脂肪等内源性大分子物质对检测结果有较大的干扰,影响结果的准确性^[32]^,因此不能忽略基质效应。

**表3:** 3种植物生长调节剂在不同基质中的线性关系、基质效应、检出限和定量限

Analyte	Sample	Linear range/(μg/L)	Linear equation	r^2^	Matrix effect	LOD/(μg/kg)	LOQ/(μg/kg)	
Chlormequat cation	pork	0.1-50	y=1.91x	0.9978	1.245	0.05	1.0	
	beef	0.1-50	y=1.76x	0.9975	1.123	0.05	0.5	
	chicken	0.1-50	y=1.79x	0.9985	0.987	0.05	0.5	
	pork liver	0.1-100	y=2.01x	0.9993	1.038	0.05	1.0	
	egg	0.1-100	y=1.89x	0.9992	1.244	0.05	0.5	
	milk	0.05-5	y=1.13x+0.0479	0.9978	1.138	0.01	0.5	
Thidiazuron	pork	0.1-50	y=3.71x-0.0561	0.9998	1.081	0.1	0.5	
	beef	0.1-50	y=3.29x+0.0434	0.9999	1.016	0.1	1.0	
	chicken	0.1-50	y=3.49x+0.0230	0.9985	0.931	0.1	1.0	
	pork liver	0.1-100	y=2.55x+0.0229	0.9997	0.871	0.1	5.0	
	egg	0.1-100	y=4.41x-0.0511	0.9998	1.276	0.1	0.5	
	milk	0.05-10	y=3.91x+0.00738	0.9965	1.053	0.05	0.5	
Paclobutrazol	pork	0.1-50	y=1.32x-0.00552	0.9987	0.491	0.05	0.5	
	beef	0.1-50	y=1.33x-0.00968	0.9999	0.380	0.05	0.5	
	chicken	0.1-50	y=1.36x-0.00930	0.9985	0.366	0.05	0.5	
	pork liver	0.1-100	y=1.62x-0.0215	0.9995	0.257	0.05	0.5	
	egg	0.1-100	y=1.61x-0.0135	0.9996	0.363	0.05	0.5	
	milk	0.05-10	y=1.66x	0.9997	0.990	0.01	0.5	

y: peak area ratio of the analyte to the isotope internal standard; x: mass concentration ratio of the analyte to the isotope internal standard.

### 2.6 线性范围、定量限和检出限

为了提高结果的准确性,本工作采用空白基质匹配内标法定量,可以有效消除基质效应。在已优化的条件下,以3种植物生长调节剂及其对应同位素内标的浓度比值为横坐标*x*,以3种植物生长调节剂及其对应同位素内标的定量离子的峰面积为纵坐标*y*,绘制标准工作曲线。不同基质中3种目标化合物在一定的浓度范围内有较好的线性关系,*r*^2^均大于0.990,详见表3。

按照1.4节方法对猪肉、牛肉、鸡肉、猪肝、鸡蛋和牛奶样品进行处理,向空白样品中添加不同水平浓度的目标化合物,以信噪比(*S/N*)≥3对应的添加水平作为检出限(LOD), *S/N*≥10对应的添加水平作为定量限(LOQ),结果见表3, 3种目标化合物在不同基质下的LOD为0.01~0.1 μg/kg, LOQ为0.5~5 μg/kg。检测中发现,噻苯隆的响应值相对较低,不同基质对噻苯隆的提取效率有较大影响,尤其在猪肝中低浓度加标回收率偏低;因此确定猪肝基质中的噻苯隆LOQ为5 μg/kg,这样既可以满足回收率的要求,同时也低于国外肝脏中MRL的最低要求。

### 2.7 回收率与精密度

选用空白样品(猪肉、牛肉、鸡肉、猪肝、鸡蛋及牛奶),分别添加LOQ、2倍LOQ和10倍LOQ 3个水平的目标物,每个添加水平进行6次实验,结果见表4。3个目标物的平均回收率为70.0%~117.4%, RSD为0.8%~16.1%,符合GB/T 27404-2008《实验室质量控制规范食品理化检测》^[33]^残留分析要求,也符合欧盟SANTE/12682/2019^[34]^的规定。

**表4:** 3种植物生长调节剂在不同基质中的回收率和相对标准偏差(*n*=6)

Matrix	Chlormequat chloride				Thidiazuron				Paclobutrazol						
	Added/(μg/kg)	Recovery/%	RSD/%		Added/(μg/kg)	Recovery/%	RSD/%		Added/(μg/kg)	Recovery/%			RSD/%		
Pork	1	75.8	2.6		0.5	115.8	3.0		0.5		86.5				8.2
	2	84.3	3.6		1	89.2	4.2		1		107.2				4.4
	10	94.5	2.5		5	104.8	3.5		5		102.1				5.5
Beef	0.5	78.9	3.4		1	74.8	6.4		0.5		77.2				4.4
	1	85.4	1.8		2	98.7	6.2		1		103.6				2.7
	5	81.9	1.9		10	102.3	12.1		5		92.2				2.3
Chicken	0.5	76.1	3.6		1	77.6	6.5		0.5		117.4				1.6
	1	92.6	2.0		2	87.3	4.1		1		98.6				6.8
	5	89.6	1.5		10	91.6	3.6		5		89.5				1.8
Pork liver	1	84.3	2.8		5	75.3	4.4		0.5		115.3				16.1
	2	80.7	3.2		10	78.1	3.9		1		95.5				5.4
	10	76.1	1.9		50	70.0	15.2		5		70.6				5.2
Egg	0.5	99.6	2.9		0.5	79.6	4.1		0.5		110.7				3.3
	1	85.9	3.4		1	106.5	6.8		1		100.4				3.4
	5	85.1	0.8		5	84.6	3.6		5		76.4				1.7
Milk	0.5	71.3	7.1		0.5	92.9	9.8		0.5		86.0				6.8
	1	91.2	7.0		1	90.1	4.7		1		91.6				3.0
	5	109.8	10.0		5	78.7	12.2		5		74.7				13.9

### 2.8 实际样品的检测

随机选取市售的动物源性食品,包括猪肉6份、牛肉5份、猪肝5份、鸡肉6份、鸡蛋10份、牛奶10份,共42份样品,运用所建立的方法对样品进行检测。结果显示,所测的10份牛奶样品中有1份牛奶检出矮壮素,结果为0.84 μg/kg,检出值低于中国、欧盟、日本等国家的MRL规定,其余样品均未检测出矮壮素、噻苯隆和多效唑。

## 3 结论

本工作建立了QuEChERS-高效液相色谱-串联质谱法测定动物源性食品中植物生长调节剂的分析方法。该方法操作简单、灵敏度高,采用基质匹配校正曲线结合内标法定量,能最大限度地消除基质干扰,使检测结果更加精确。方法定量限满足不同国家的最低限量要求,适用于大批量样品的检测工作,可为进出口动物源性食品的植物生长调节剂类农药的风险监控提供检测技术。由于同位素内标物质价格高且不易获得,本工作只对植物生长调节剂类中的3种农药进行了讨论研究,但也为动物源性食品中其他PGRs农药残留提供了参考。
